# Nephrolithiasis and Nephrocalcinosis in Childhood—Risk Factor-Related Current and Future Treatment Options

**DOI:** 10.3389/fped.2018.00098

**Published:** 2018-04-12

**Authors:** Alexander Weigert, Bernd Hoppe

**Affiliations:** Division of Pediatric Nephrology, Department of Pediatrics, University Children’s Hospital, Bonn, Germany

**Keywords:** nephrolithiasis, urolithiasis, nephrocalcinosis, treatment, therapy

## Abstract

Nephrolithiasis, urolithiasis, and nephrocalcinosis (NC) have become common causes of hospitalization and referral to pediatric outpatient clinics. It is of utmost importance to start with diagnostic evaluation directly after the first passage of a kidney stone, or if NC is diagnosed, in each pediatric patient. This is necessary, as in about 80% of children a metabolic reason for stone disease is detected. Current treatment options are scarce and mainly include general measures like an increased fluid intake or elevating the solubility of a lithogenic substance. According to the given lithogenic risk factor(s), specific treatment options are available and are being summarized in this review. Furthermore, an outlook on potential future treatment options, including innovative strategies such as mRNA-based or recombinant enzyme substitution therapy, is given.

## Introduction

Nephrolithiasis (NL) and urolithiasis (UL) describe solid stones appearing in the kidney (NL) or in the lower urinary tract (UL). The term nephrocalcinosis (NC) expresses deposits of calcium salts within the renal tubules, the tubular epithelium, and/or the interstitium (1). NC is also classified ultrasonographically due to the anatomic area involved: cortical and diffuse NC or medullary NC, respectively, the latter subdivided according to the degree of echogenicity as medullary NC grade I-III (2). All three entities have become common causes of hospitalization and presentation in pediatric outpatient clinics (3). Although exact numbers for prevalence and incidence rates are still not known, it is said, that numbers increase in pediatrics, as it was shown in the adult population. For example, in the US, the incidence of NL is estimated to be 36–57 per 100,000 population (4). Since in up to 40% of children the diagnosis is made incidentally (for example, after a first or recurrent urinary tract infection) due to the high proportion of unspecific symptoms, the accurate incidence might be underestimated.

Nephrolithiasis affects children of all ages. During the first decade of life, boys are more frequently prone to develop NL, while girls are more frequently observed to develop kidney stones in the second decade of life (5). Clinical presentation is highly variable and depends on the age of affected children, e.g., failure to thrive in infants, or typical flank pain in the older child/adolescent patient (6). If present, newly diagnosed microhematuria or macrohematuria can be the first clinical sign for NL.

Decision-making on treatment regimens should be based on thorough evaluation of the underlying risk factors, as metabolic disorders are found in ~80% of children with kidney stones/NC (7). Diagnostic evaluation thus includes examination of metabolic disorders leading to elevated urinary excretion of a lithogenic factor, or to decreased excretion of an anti-lithogenic substance, but also anatomical abnormalities, urinary tract infections and prematurity, insufficient fluid uptake (8), and, with increasing importance, obesity (9). The 24-h urine is the most important tool in diagnostic workup of NL/NC, since blood examination usually remains unremarkable.

Overall, treatment options are scarce and include general measures such as increased fluid intake and a balanced diet with avoidance of excessive sodium intake. According to the given lithogenic risk factor(s), specific treatment options are already available. However, the current armament to treat the (pediatric) patient with a metabolic disease, thereby resulting in severe risk of kidney stone development or progressive NC, is minor.

## General Risk Factors

Dietary excesses, e.g., for oxalate containing food, or simply as hypercaloric diet (“metabolic syndrome”), may also influence the development of NL. While increased sodium intake can lead to an increased urinary calcium excretion, low calcium diet might promote increased intestinal oxalate absorption and thus resulting in secondary hyperoxaluria (10, 11).

### General Measures

#### Fluid and Diet

A MUST for all stone patients is a high fluid intake: more than 1.5–2 L × 1.73 m^2^ body surface area per day, distributed over the entire day, is recommended. This prevents peaks of high concentration levels of a given soluble. However, dietary recommendations should be handled carefully. Excessive dietary sodium uptake has to be avoided, as it promotes calcium excretion (12). Calcium restriction can lead to an increased intestinal oxalate absorption and hence urinary oxalate excretion. Because of that and since calcium restriction might lead to low bone mineral density, this is an obsolete procedure in patients with hypercalciuria (13). Protein restriction, as well as excessive protein uptake, should of course be avoided, as it leads to hypercalciuria and hypocitraturia, due to an increased acid load. Keep in mind (in the pediatric patient) that restriction of protein includes the risk of growth retardation! A diet including the risk of metabolic syndrome (high fat and fructose intake) showed an increased risk of NL in epidemiologic studies (14). Vegetables and fruits provide a good source of citrate and potassium (both stone inhibitors) and by that decrease the risk of NL (15). Summing up, children with the risk of NL should stick to a normal and balanced diet.

#### Crystallization Inhibitors

Citrate and magnesium effectively increase urinary solubility especially of cystine, calcium oxalate and uric acid at given urinary pH levels (uric acid and calcium oxalate > 6.2 < 7.4, cysteine >8). Citrate is metabolized in the liver into bicarbonate, which elevates urinary pH, resulting in a reduced tubular citrate reabsorption. Urinary citrate reduces calcium excretion by 30% and it binds to urinary calcium forming a soluble complex, which reduces the precipitation of calcium with other lithogenic substances (16). Citrate is best delivered as potassium citrate or as sodium potassium citrate. The adequate increase of citrate excretion can be achieved by a dosage of 0.1–0.2 g/kg body weight (0.3–0.6 mmol/kg). Patients with distal RTA usually require higher dosages (0.2–0.3 g/kg body weight), and the dosage has to be adapted to urine pH (17). Since very high urinary pH levels promote calcium phosphate precipitation, urine alkalization therapy has to be monitored!

## Hypercalciuria

The most common risk factor for NL in childhood is hypercalciuria (18), which can arise from idiopathic hypercalciuria (19), a multifactorial disease, as well as from genetic disorders (7) or other underlying diseases, such as (primary) hyperparathyroidism.

An example for a genetic reason (for more information, see Table S1 in Supplementary Material) is autosomal dominant hypocalcemic hypercalciuria (ADHH), which is caused by mutations in the calcium-sensing receptor (CaSR) gene, and genes connected to that receptor pathway (Gα11). Electrolytes in these patients show elevated serum phosphate along with low serum calcium and magnesium (20). ADHH is associated with calcification of brain and kidney and with cataract. Other genetic diseases resulting in hypercalciuria is familial hypomagnesemia hypercalciuria and nephrocalcinosis syndrome or are the different types of Dent Disease (Dent 1 and 2). In the latter, a diagnostic hint can be male gender (x-chromosomal recessive) accompanied by low molecular weight proteinuria and severe hypercalciuria (21).

Secondary hypercalciuria can result from medication (e.g., furosemide, vitamin D) or parenteral nutrition [higher daily intake of protein, sodium, phosphorus, and ascorbic acids, leading to significantly increased urinary calcium and oxalate, but lowish citrate excretion and hence urinary calcium oxalate supersaturation (22)].

### Current Treatment

When severe hypercalciuria is present, thiazides reduce the urinary calcium excretion, since they increase calcium reabsorption in the distal and proximal tubule (23). Thiazides also are capable of encountering a reduced bone density in patients with hypercalciuria (24). Daily dosage is 0.5–1 mg/kg body weight, twice a day. Side effects include hypotension and hypokalemia. In case of hypokalemia, amiloride (a potassium-sparing and calcium-lowering diuretic) should be added (25).

### Future Treatment Options

#### Experimental (Animal) Studies

In knock-in mutant mice of the calcium-sensing receptor (CaSR), which mimics ADHH, calcilytic therapy has been shown to reduce urinary calcium excretion, which was able to prevent renal calcification. Calcilytics are CaSR antagonists capable of improving serum calcium and phosphate, due to stimulating PTH secretion (26).

In Dent disease, first experience with bone marrow transplantation in Clcn5 knockout mice, a model for Dent disease, showed an improvement of protein-, calci-, and glucosuria as well as reduced polyuria. This most likely results from bone marrow-derived cells engaged in kidney interstitium (27).

Recently, Rendu et al. were able to restore normal mRNA and protein levels in fibroblasts of a patient with a deep intronic mutation in the OCRL gene (Lowe syndrome, Dent II), showing RNA-based [mRNA or RNA interference (RNAi)] therapy might be a future approach in these patients (28).

#### Case Series

For patients with CYP24A1 mutations, leading to idiopathic infantile hypercalcemia (IIH, Table S1 in Supplementary Material), single reports of treatment with rifampicin have been published. Rifampicin acts as a potent inductor of CYP3A4, an inactivator of many xenobiotics, and it was shown that it is capable of reducing urinary calcium excretion, serum calcium levels, and 1,25 (OH)2 D3 (29).

## Hyperoxaluria

There are three types of genetic hyperoxaluria: type I (PH I), secondary to mutation in the alanine/glyoxylate aminotransferase (AGT), is characterized through increased urinary excretion of oxalate and glycolate (30), type II (PH II), secondary to mutation in the glyoxylate/hydroxypyruvate reductase (GRHPR), is characterized by elevated oxalate and l-glyceric acid urinary excretion (31) and type III (PH III), secondary to mutation in the 4-hydroxy-2-oxoglutarate aldolase (HOGA 1), characterized by raised urinary excretion of oxalate and hydroxy-oxo-glutarate and/or hydroxy-oxo-glutamate (32). Secondary hyperoxaluria results from an increased intestinal oxalate uptake, due to malabsorptive states such as short bowel disease or chronic inflammatory bowel disease, by increased dietary oxalate intake, or lack of intestinal oxalate degrading bacteria (33).

### Current Treatment

There are no approved drugs for the treatment of primary hyperoxaluria (PH). Since most of the oxalate is produced endogenously by the liver, an oxalate-restricted diet is often of limited use to these patients. Treatment in supra-physiological doses (5–20 mg/kg body weight per day) of pyridoxal-phosphate (vitamin B6), as the cofactor for the defective enzyme AGT in type I PH, reduces the endogenous oxalate production and the urinary excretion in about one-third of all PH I patients, especially those with missense mutations (34). Known side effects are polyneuropathy, acne and bullous skin eruptions. For all other PH I and those patients with PH types II and III the current measures, high fluid intake and citrate medication are the only further treatment possibilities.

Patients with PH (I) and end-stage renal failure (ESRF) should be transplanted as soon as possible since no renal replacement therapy is able to remove oxalate adequately. In PH I, combined liver and kidney, or two-timed transplantation, e.g., kidney after liver, are recommended based on the systemic oxalate burden of the patient. Patients with PH II shall only receive kidney transplantation since the defect enzyme is ubiquitous (7). However, just recently a combined liver–kidney transplantation was reported in a PH II patient (35). In PH III only, one patient with ESRF was so far reported (PH III seems to be the mildest and therefore easiest to handle type of PH); hence, transplant strategies were not yet established (36).

### Future Treatment Options

#### Experimental (Animal) Studies

**Figure 1:**
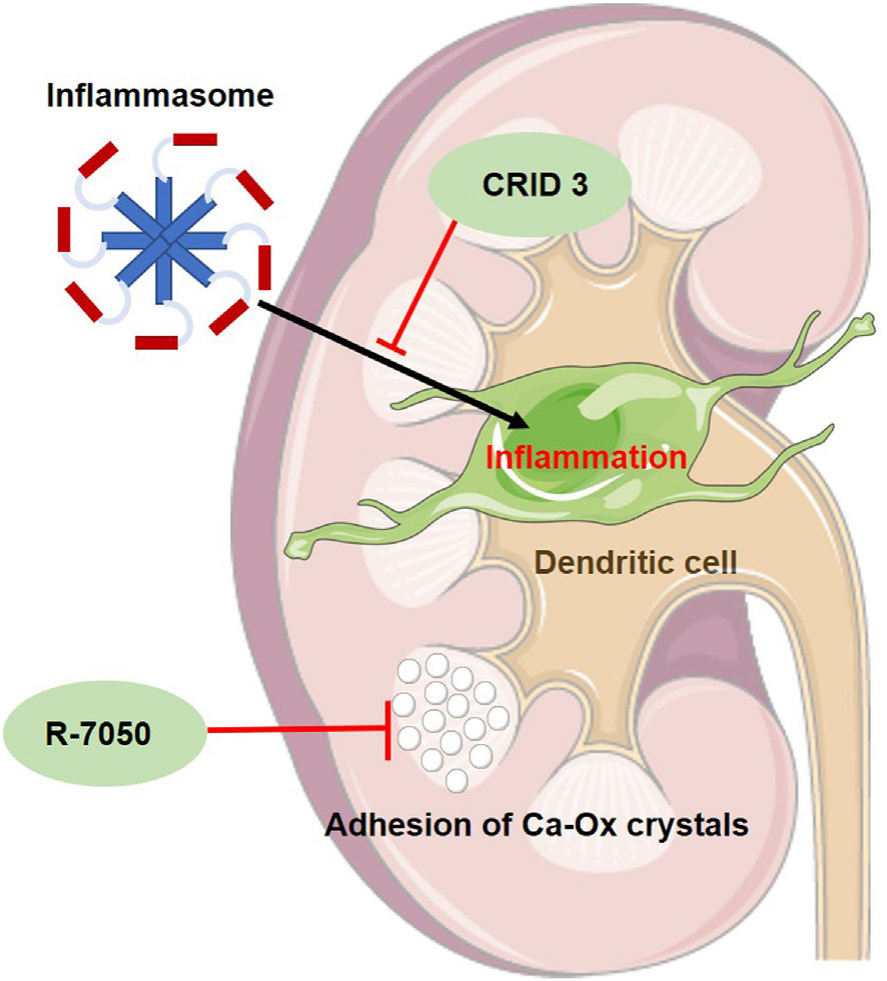
Schematic figure of the mode of action of two new experimental drugs for primary hyperoxaluria I. CRID 3 inhibits inflammasome-mediated inflammation in dendritic cells of the kidney and thus reducing kidney fibrosis. R-7050 is a TNF-receptor inhibitor, delaying the progression of nephrocalcinosis since it seems to prevent adhesion of calcium oxalate (Ca-Ox) crystals to renal tubules.

In hyperoxaluric mice, the TNF receptor inhibitor R-7050 was able to delay the progression of NC. The TNFR signal pathway seems to be essential in the adhesion of calcium oxalate crystals (see Figure 1) to the luminal membrane of renal tubules (37).

A currently identified and obviously major player in oxalate-induced kidney inflammation is the activation of the inflammasome pathway (38). The inflammasome is a protein complex, which, when activated, activates IL-1β and IL-18 production and thus promoting local inflammation (see Figure 1). In mice with crystal-induced kidney fibrosis, which was induced by an oxalate or adenine-rich diet, a specific inhibitor, CP-456,773 (or CRID3), of the NLRP3 inflammasome pathway was able to delay the progress of kidney fibrosis (39).

**Figure 2:**
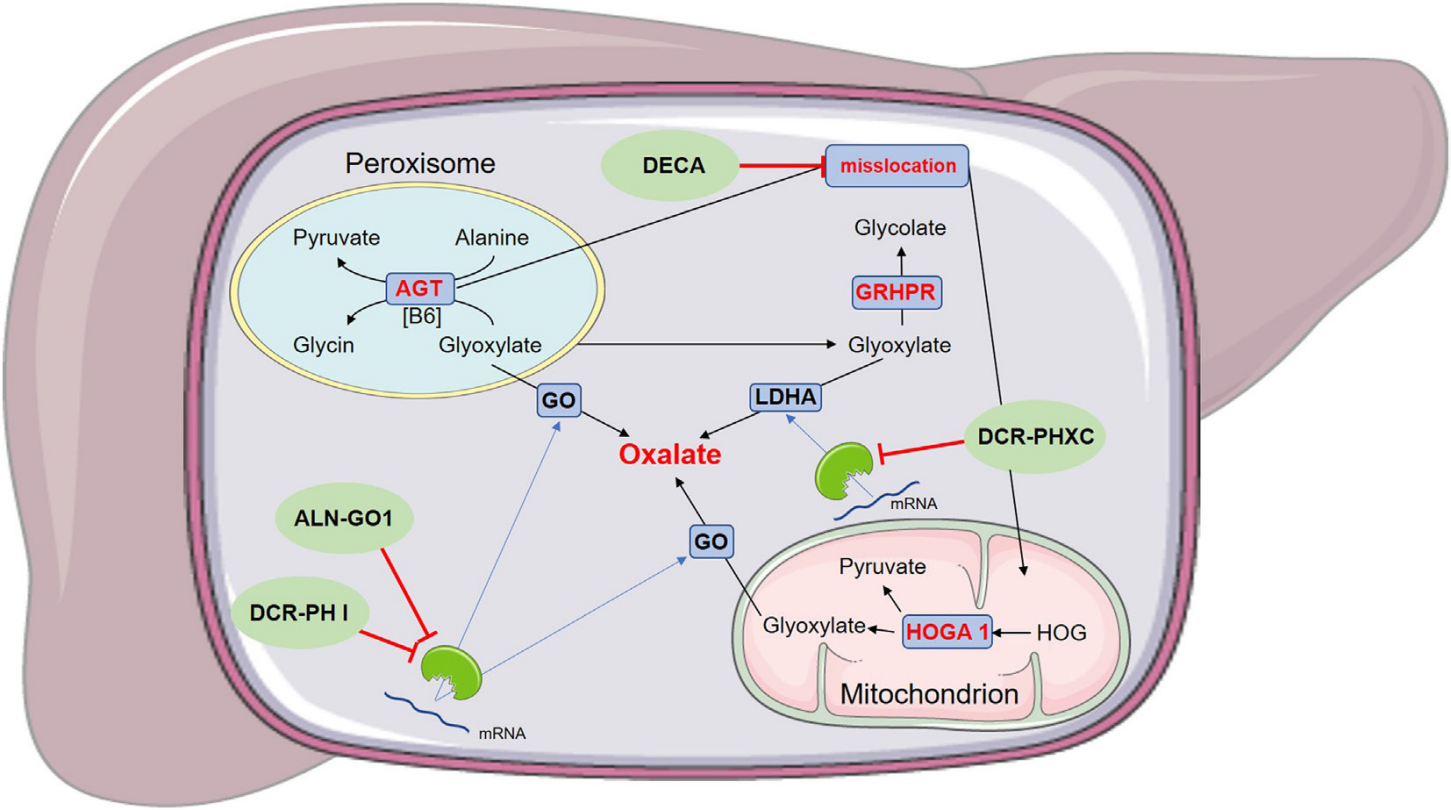
Schematic figure of the mode of action of experimental drugs for primary hyperoxaluria (PH) I. ALN-GO1 and DCR-PH I are RNA interference (RNAi)-based drugs, preventing the translation of glycolate oxidase (GO) and thus reducing endogenous oxalate production. DCR-PHXC as well is an RNAi-based drug targeting the liver-specific lactate dehydrogenase A (LDHA), also reducing endogenous oxalate production. Dequalinium chloride (DECA) prevents the misslocation of alanine:glyoxylate aminotransferase (AGT). This is only applicable for a certain PH I mutation (P11LG170R allele), since other mutations do not cause a misslocation of AGT.

In a certain PH I mutation (P11LG170R allele), the functional AGT is mistargeted into mitochondria instead into peroxisomes. Dequalinium chloride (DECA) inhibits mitochondrial protein import into the mitochondrium and restores transportation of AGT into the peroxisome, where it regains its regular function (see Figure 2). This reduces oxalate accumulation, similar to pyridoxal phosphate and even has additive effects with that therapy (40). If other AGT variations lead to a mislocation of the enzyme and could, therefore, be applicable for DECA therapy, is not known yet.

#### Ongoing Human Clinical Trials

**Figure 3:**
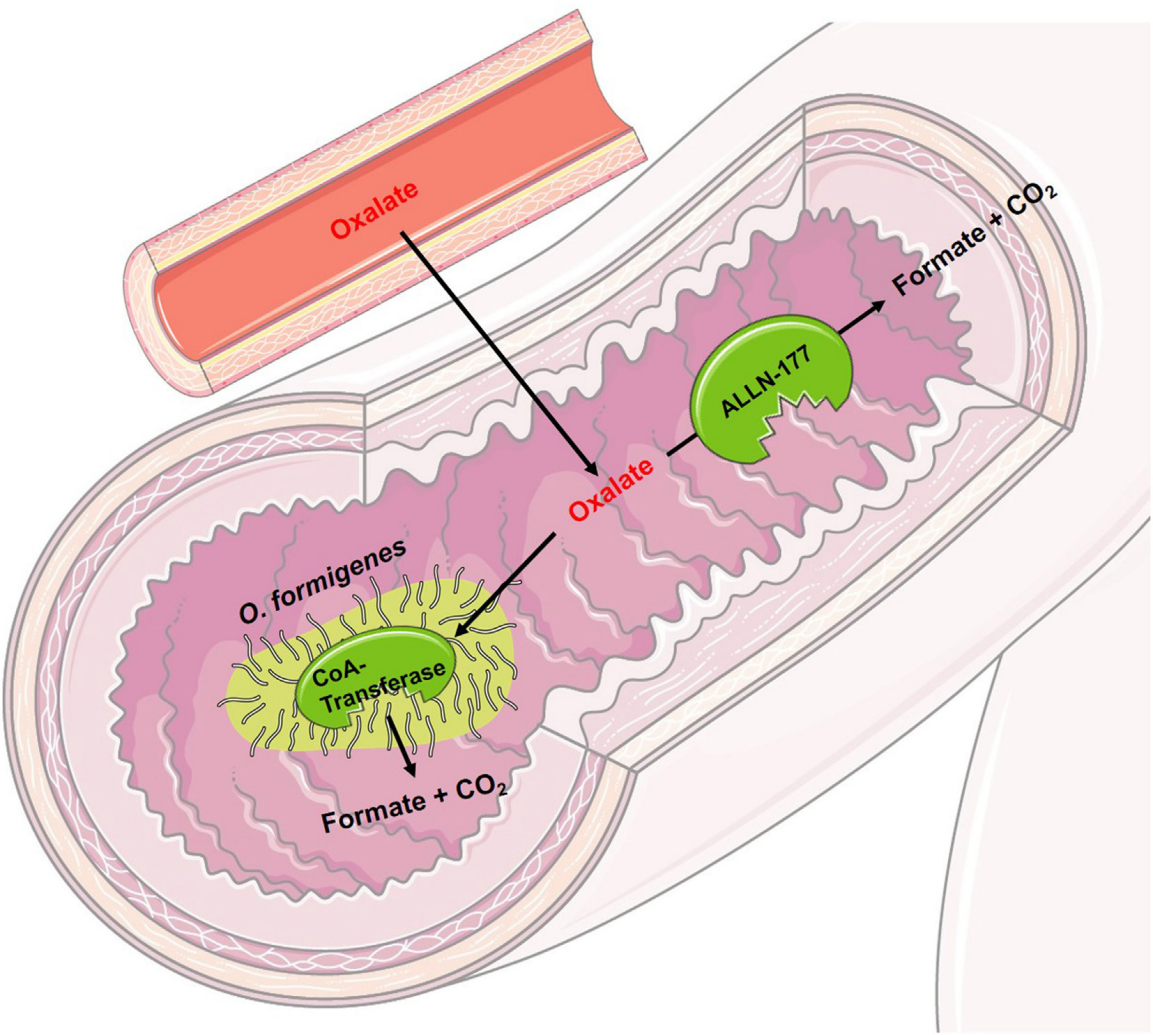
Schematic figure of the mode of operation of experimental drugs for primary hyperoxaluria I. *Oxalobacter formigenes* uses oxalate as its sole carbon source. Orally administered, it degrades intraluminal oxalate in the intestine. By a concentration gradient and through activation of the intestinal oxalate transporter, oxalate is transported from the blood into the intestinal lumen. ALLN-177 is a recombined, microbial oxalate decarboxylase leading to the same intraluminal effect as *O. Formigenes*, however, maybe unable to increase the shift of blood oxalate into the intestinal lumen, as it cannot directly activate the intestinal oxalate transporter.

Oral therapy with *Oxalobacter formigenes* (Oxabact, Oxthera AB, Sweden), an anaerobic bacterium that degrades oxalate for its sole carbon source (41), promotes the removal of endogenously produced oxalate *via* the intestinal tract (see Figure 3). Activation of the intestinal oxalate transporter and a high concentration gradient from blood to intestinal lumen induces an oxalate shift with the possibility that oxalate can be metabolized by intestinal *Oxalobacter*. First, data showed conflicting results. A Phase I study led to a significant reduction in urinary oxalate excretion (42), whereas other studies over a longer period of time showed no significant results (43, 44). Since the interpersonal effect of *O. formigenes* seems to be variable, and especially depending on patient’s compliance, further studies according to the efficacy of *O. formigenes* are currently being conducted. Nevertheless, *ad hoc* interpretation of study results showed a positive effect on kidney function over time. In addition, all recent *Oxalobacter* trials made obvious that urinary oxalate excretion might not be the perfect endpoint for a treatment study in patients with PH. Therefore, a further study will evaluate a variety of parameters, mostly focusing on plasma oxalate follow-up and amelioration or prevention of systemic oxalate deposition. A study with PH I patients on maintenance hemodialysis is ongoing and preliminary results show improvement of plasma oxalate levels, as well of systemic oxalate burden of those patients being compliant.

ALLN-177 (Allena Pharmaceuticals, USA) is a recombinant, microbial enzymatic oxalate decarboxylase, which degrades oxalate in the gastrointestinal tract (see Figure 3). It has been shown that ALLN-177 is able to reduce the urinary oxalate excretion in healthy persons (45). If ALLN-177 can help patients with primary or secondary hyperoxaluria is currently under investigation. Degrading of dietary oxalate will obviously be possible; however, removal of endogenously produced oxalate *via* the intestinal tract might be tricky to achieve with such a medication. On overt, concentration gradient of oxalate (blood vs. intestinal tract) might lead to secretion of oxalate into the intestinal lumen; however, an activation of the intestinal oxalate transporter, as it is seen with *Oxalobacter* treatment, is still under debate.

Another therapeutic approach is the administration of ALN-GO1 (Alnylam Pharmaceuticals, USA), an investigational RNAi medication. RNAi function is based on small RNA molecules (small interfering RNA, siRNAi), which bind to cytoplasmatic enzymes and form a highly specific working complex, that decomposes mRNA and thus prevents the translation of that mRNA into the subsequent protein (46). ALN-GO1 targets the glycolate oxidase (GO) mRNA (see Figure 2), preventing the translation from mRNA into the working protein and thus reducing the development of glyoxylate and hence the production of oxalate. A study on animals showed a reduction of urinary oxalate excretion in mice and non-human primates by up to 98%, after multiple subcutaneous administrations (47). Initial results of a phase I Study of ALN-GO1, as presented at the 17th Congress of the International Pediatric Nephrology Association (IPNA), ALN-GO1 was able to silence up to 80% of the GO mRNA, without serious adverse events in healthy subjects (48). Preliminary results of the ongoing phase I/II study of ALN-GO1, presented at the American Society of Nephrology (ASN) annual meeting in 2017, showed a reduction of urinary oxalate excretion, up to 50%, in PH I patients without treatment-related serious adverse events (48).

Another RNAi, which was initially investigated in a phase I study, is DCR-PH1 (Dicerna Pharmaceuticals, USA). DCR-PH1 also prevents the translation of GO (see Figure 2). After having shown its capability of reducing urinary oxalate in animal models, both healthy volunteers and human patients with PH I are now being enrolled in a Phase II study (49), which was later interrupted. Dicerna Pharmaceuticals just recently applied for a phase I study of DCR-PHXC, another RNAi-based therapy, targeting the lactate dehydrogenase A (LDHA) (see Figure 2). In animal models, DCR-PHXC was able to silence the liver LDHA and thus preventing an excessive oxalate production (50). This medication would hence be able for treatment of patients with all types of PH.

## Cystinuria

Cystinuria, one of the most frequent autosomal-recessive inherited genetic disorders (prevalence: 1:7,000), is responsible for about 5–10% of all pediatric kidney stones. A defective tubular reabsorption leads to an increased urinary excretion of the dibasic amino acids cystine, ornithine, lysine, and arginine, but only cystine is able to promote stone formation since the other acids are highly soluble in urine (51).

### Current Treatment

The main goal of therapy for patients with cystinuria is urine alkalization. Cystine has a higher solubility at a pH above 8. Chelating agents such as d-penicillamine and alphamercaptopropionyl-glycine (MPG) destroy the bond between two cysteine molecules, which separately have a higher solubility than combined with cystine. Both are equally effective and should be administered with 20–40 mg/kg body weight (52). Side effects include rash, exanthema, arthralgia, thrombocytopenia, polymyositis, and nephritic syndrome. MPG seems to have fewer side effects than d-penicillamine (53), which furthermore reduces the level of pyridoxine and therefore has to be replaced during therapy. The ACE inhibitor captopril has a similar effect like MPG, but fewer side effects (54). A urinary cystine excretion above 720 mg/day justifies a treatment with 75–150 mg of captopril per day, although the effect is variable (55). Ascorbic acid in high doses (3–5 g/day) not only can decrease the urinary cystine excretion but can increase endogenous oxalate production and therefore urinary oxalate excretion, so no clear dosage recommendation can be given for that therapy (56). A careful protein reduced diet is recommended since the contained methionine is metabolized to cystine.

### Future Treatment Options

#### Experimental (Animal) Studies

In cystinuria, several substances have been examined on their ability to inhibit cystine crystal growth. One of the first was l-cystine dimethyl ester (CDME), which was able to reduce cystine stone size, but not urinary cystine excretion in a knockout mouse model (57). The l-cystine diamides l-cystine bismorpholide and l-cystine bis (*N*′-methylpiperazide) were *in vitro* more powerful in increasing cystine solubility, than CDME. l-cystine bis (*N*′-methylpiperazide) has shown its ability to reduce stone formation in cystinuria knockout mice and thus providing a possible new treatment option in cystinuria (58).

Another substance that was able to increase cystine solubility in a knockout mouse model is α-lipoic acid. Zee et al. report a reduction of stone formation due to α-lipoic acid as nutritional supplementation (59). The effect of α-lipoic acid on kidney stone recurrence is now being evaluated in a human clinical trial (NCT02910531) (60).

#### Ongoing Human Clinical Trials

A currently recruiting phase II trial is investigating the safety and effectiveness of bucillamine (NCT02942420). Bucillamine is a drug developed from tioprinin, currently used as an antirheumatic agent and, acting as a Thiol donor, which might be capable of binding cysteine from urine and thus reducing the risk of stone formation (61).

A pilot study (NCT02538016) on the effect and safety of tolvaptan, a vasopressin antagonist, is also currently being conducted (62).

## Purine Stones

Purine stones can result from hyperuricosuria secondary to tumor lysis syndrome, or rarer, from genetic defects (e.g., Lesch–Nyhan syndrome), or enzymatic defects such as 2,8-dihydroxadeninuria (63) or xanthinuria, which is based on xanthine oxidase deficiency (64).

### Current Treatment (Uric Acid, 2,8-Dihydroxyadenine, Xanthine)

Uric acid stones can also be treated best with urine alkalinization. Urine pH should be kept above 6.5 and excessive protein intake should be avoided. In severe cases, Allopurinol, an inhibitor of xanthine oxidase, can be applied, since it reduces serum uric acid. Dosage must be carefully triggered since it can lead to relevant xanthinuria. Fluid intake, leading to a urinary output of at least 2–3 l per day is recommended (65). Allopurinol also can be used in 2,8-Dihydroxyadeninuria, as well as hyperhydration and dietary restriction of adenine and purine. If allopurinol is not applicable, e.g., due to allergic reactions, single cases with successive treatment with febuxostat have been reported (66) and clinical experience supports these reports.

Patients suffering from xanthinuria do not benefit from urine alkalization. Increased fluid uptake still remains the only effective therapeutic measure (64).

## Infectious Stones

Urinary tract infections with urease-producing bacteria (e.g., proteus species) lead to the formation of struvite stones. The bacteria are capable of hydrolyzing ammonia into ammonium ions, resulting in an elevated urinary pH (67). This promotes the formation of carbonate ions and the production of trivalent phosphate ions, both major components of struvite stones.

### Current Treatment

Children suffering from infectious stones must be treated with adequate antibiotics. Formatted stones require an extraction procedure. If the stone stays *in situ*, it provides an optimal nidus for bacterial growth and therefore increases the risk of re-infection.

## Hypocitraturia

Hypocitraturia is known to be a common risk factor in preterm infants (68). In older children, it is most prevalent in some parts of the world (e.g., Turkey). It is also a characteristic finding in complete distal renal tubular acidosis (d-RTA) and can be found in patients with metabolic acidosis, hypokalemia, urinary tract infections, and malabsorption syndromes (69).

### Current Treatment

Hypocitraturia can be encountered by giving potassium citrate (1 mEq/kg daily). This treatment successfully reduces stone reoccurrence (70). Since hypocitraturia is often accompanied by other abnormal urine findings, these must be treated according to their entity.

## Renal Tubular Acidosis (RTA)

Renal tubular acidosis is characterized by an impaired H^+^ excretion, hypercalciuria, and hypocitraturia (17). This leads to early onset of NL and is accompanied by loss of hearing. Incomplete distal RTA causes kidney stones without clear acidosis being present.

Nephrolithiasis in children is seldom purely drug related (71), but certain drugs increase the risk of NL. Two pathomechanisms might lead to NL: drugs excreted by the kidney with poor solubility (e.g., indinavir, acyclovir, TMP or sulfadiazine) can either provide a direct nidus for stone formation (72) or increase the excretion of urinary lithogenic substances (e.g., furosemide induces hypercalciuria in preterm infants, carboanhydrase inhibitors result in hypocitraturia and hypercalciuria) (73).

### Current Treatment

The continuous supplementation of alkali is the only therapeutic approach in all forms of RTA. This can be achieved by administration of sodium and potassium bicarbonate, or citrate salts. Therapeutic goal is a serum bicarbonate above 20 mEq/L in infants an >22 mEq/L in children, keeping in mind the amount of required alkali decreases with coming of age: 5–8 mEq/kg/day for infants, 3–4 mEq/kg/day for children, and 1–2 mEq/kg/day for adults (74).

### Future Treatment Options

#### Ongoing Human Clinical Trials

Six-month data presented at the ASN annual meeting in 2017 showed promising results of a phase III trial for ADV7103 (bicarbonate and citrate in 2-mm granules), a new agent in the treatment of dRTA. ADV7103 is a slow release medication of alkaline citrate, which provides equilibrate alkali dosing over 12 h. A dosage of ADV7103 twice a day was able to maintain a normal blood bicarbonate level and improved quality of life in patients with dRTA (75). This is in contrast to the current alkaline medication, where both blood bicarbonate, as well as urine citrate excretion may fluctuate over the day and depend much more on the timing of medication (here >2 times a day). Advicenne pharmaceuticals is now seeking market authorization for ADV7103 and was granted orphan drug designation already by the EU in 06/2017. In addition to the potential treatment in dRTA, a phase II/III trial for the use of ADV7103 in patients with cystinuria is currently being initiated (76).

## Conclusion and Outlook

The current therapeutic possibilities for patients with NL or NC are minor, promising research is on the rise to evaluate new treatment options. They range from therapeutic manipulation of a substance’ urine solubility (e.g., in cystinuria), mRNA-based approaches (e.g., PH and Lowe syndrome) to soluble degrading enzymes or bacteria (in PH). Many of these therapeutical chances have only been tested *in vitro* or in animal models. The way to get them into human use still may be long, but patients’ distress is urging scientist to proceed with their work (20, 21, 77–93).

## Author Contributions

Both authors contributed equally to this paper.

## Conflict of Interest Statement

The authors declare that the research was conducted in the absence of any commercial or financial relationships that could be construed as a potential conflict of interest.
