# Supplementary material for: Nephrolithiasis and Nephrocalcinosis in Childhood—Risk Factor-Related Current and Future Treatment Options
Source: Front Pediatr. 2018 Apr 12;6:98. doi: 10.3389/fped.2018.00098 (PMC5906718; doi:10.3389/fped.2018.00098)
Supplement: Supplementary file 1 [file Table_1.DOCX]

**Table 1 I Risk factors for nephrolithiasis with accompanying clinic and current therapy options. (modified according to Habbig et al., [10])**

| **Risk factor/entity** | **Clinic** | **Current therapy options** | **Future therapeutic approaches** | **Good to know** | |
| --- | --- | --- | --- | --- | --- |
| **1. Hypercalciuria** | | | | | |
| idiopathic hypercalciuria [11] | normocalcemia, normal PTH levels, low bone mineral density | thiazide diuretics |  |  | |
| idiopathic infantile hypercalcemia ‘IIH‘ [72] | hypercalcemia, hypercalciuria, NC, failure to thrive, hypotonia, lethargy | low calcium and vitamin D diet, loop/thiazide diuretics, ketoconazole, in acute settings: bisphosphonates, glucocorticoids (limited effect) | rifampicin [54] (case reports) | manifestation in adults follows less severe course of disease | |
| autosomal dominant hypocalcemic hypercalciuria ’ADHH’ [12] | asymptomatic hypocalcemia, serum phosphate elevated, serum magnesium low, PTH in low normal range, tissue calcification of brain and kidney, cataract | calcitriol, thiazide, recombinant parathormone | calcilytics [51] (animal studies) | vitamin D substitution will worsen hypercalciuria, resulting in NC, NL and eventually ESRF | |
| Dent’s disease [13]   - Dent 1      - Dent 2 | Fanconi syndrome (FS) with aminoaciduria, phosphaturia, glycosuria, kaliuresis, impaired acidification. LMW proteinuria, less severe hypercalciuria with age, NC + UL and regular CRF  mental retardation, hypotonia, rickets, proximal tubular defects, nephrotic range proteinuria, metabolic acidosis, hypercalciuria, NC + UL, regular CRF | thiazide, phosphate supplementation, enalapril  “ | bone marrow transplantation [52] (animal studies)  RNA-based therapy [53] (experimental) | male gender (x-chromosomal recessive)  male gender (x-chromosomal recessive) | |
| Lowe’s (oculorenocerebral) syndome [75] | like Dent 2, but with additional congenital cataract and muscular hypotonia | symptomatic (e.g. citrate, bicarbonate, phosphate supplementation, cataract surgery) | RNA-based therapy [53] (experimental) | life span rarely exceeds 40 years | |
| Bartter syndrome (BS) [88]   - type 1      - type 2 - type 3      - type 4      - type 5 | - classic BS: Hypokalemic alkalosis, renal salt wasting, hypercalciuria, elevated serum renin, secondary hyperaldosteronism, hyperprostaglandinemia, NC, risk of CRF  - antenatal BS: polyhydramnios, renal salt wasting, prematurity, volume depletion  hypercalciuria and NC, classic and antenatal BS  classical BS with wide phenotype variation, less hypercalciuria and NC, risk of CRF  usually severe antenatal BS with deafness, but less hypercalciuria and NC  early symptomatic hypocalcemia and hypercalciuria with NC, followed by classical BS features | potassium supplementation, ACE inhibitors and Angiotensin receptor blockers, amiloride, spironolactone, indomethacin, celecoxib  “  “  “  “ |  |  | |
| hereditary hypophosphatemic rickets with hypercalciuria [76] | excessive excretion of urinary phosphate, hypophosphatemia, severe rickets, hypercalciuria without hypercalcemia, UL | phosphate supplementation |  |  | |
| Williams-Beuren syndrome [80] | mental retardation, distinctive neuropsychological profile (‘happy party manner‘), cardiovascular anomalies and anomalies of the urinary tract, temporary hypercalcemia and -uria, NC and NL | highly individual due to variable mortalities |  | mostly sporadic, risk of NC 5 – 10 % | |
| familial hypomagnesemia with hypercalciuria and nephrocalcinosis ‘FHHNC‘ [78] | symptomatic hypomagnesemia and hypocalcemia, hypercalciuria with NC, NL and regular CRF | thiazides, magnesium supplementation |  |  | |
| Wilson’s disease [79] | Fanconi syndrome (see Dent 1), liver dysfunction, neurological symptoms, Kyser-Fleischer corneal ring, urinary copper excretion elevated, reduced ceruloplasmin, hypercalciuria with NC, UL and CRF | low copper diet, trientine, penicillamine, liver transplantation |  |  | |
| tyrosinemia type 1 [81] | Fanconi syndrome (see Dent 1), rickets, liver failure, coagulopathy, hypercalciuria with NC, UL and CRF | nitisinone, low tyrosin and phenylalanine diet, liver transplantation |  | connected with liver disease of varying extend | |
| Liddle’s syndrome/ pseudohyperaldosteronism type 1 [82] | triad: hypokalemia, alkalosis and sodium-sensitive hypertension. Suppressed aldosterone, hypercalciuria with NC and risk of CRF | amiloride |  |  | |
| Gordon’s syndrome/ pseudohyperaldosteronism type 2 [83] | hyperkalemia, reduced ammonium excretion resulting in metabolic acidosis, hypertension and hypercalciuria | thiazide |  |  | |
| primary hyperparathyroidism | frequently associated with hypercalciuria. | thiazide diuretics, parathyroidectomy |  |  | |
| hypervitaminosis D [73] | hypercalcuria appears before hypercalcemia, lethargy, confusion, in extreme cases stupor, obstipation, polyuria | withhold vitamin d medication, increased fluid intake, loop diuretics, glucocorticoids |  |  | |
| granulaomatosus disease   - Sarcoidosis/tuberculosis - Lymphomas | 10% of patients with variable hypercalciuria  similar to hypervitaminosis D | glucocorticoids, ketoconazole  “ |  | present hypercalcemia associated with reduced survival | |
| **2. Cystinuria** | | | | | |
| cystinuria type I and II, mixed type I/II phenotype [17] | defective reabsorption of cystine, lysine, ornithine and arginine. Only cystein forms stones. Often bilateral NL and high risk of recurrent stones | urine alkalinization, D-penicillamine, alpha-mercaptopropionyl-glycine (MPG), ACE-inhibitors, Captopril, high dosed ascorbic acid | - L-cystine dimethyl ester (CDME) [55] (animal studies) - L-cystine bismorpholide [56] (animal studies)  - L-cystine bis (N’-methylpiperazide) [56] (animal studies) - α-lipoic acid [58] (animal studies) | | Ascorbic acid can increase urinary oxalate excretion |
| **3. Hyperoxaluria** | | | | | |
| primary hyperoxaluria   - type I (PH I) [18] - type II (PH II) [19] - type III (PH III) [20] | progressive NC and recurrent NL, severe hyperoxaluria, hyperglycemic aciduria, systemic oxalate depositions leading to multisystemic disease character, regular ESRF (from neonatal to late adulthood)  NC less frequent, recurrent NL, hyperoxaluria with L-glyceric aciduria, lower risk of ESRF (approx. 20%)  recurrent NL in childhood, seems to remit with age, so far only one ESRF has been reported | pyridoxal phosphate, combined/two timed liver and kidney transplantation  kidney transplantation  increased fluid uptake | PH I: - enzyme substitution: ALLN-177 [66] (currently in clinical trials)  - RNA based therapy: ALN-GO1 [67]; DCR-PH1 [70]; DCR-PHXC [71] (currently in clinical trials  - Dequalinium chloride/DECA [61] (animal studies)  all PH forms:  *- Oxalobacter formigenes* [63] (currently in clinical trials) - inflammasome-inhibition CRID-3 [60] (animal studies) - TNF inhibition R-7050 [58] (animal studies) | | ~80% of PH cases  < 10% of PH cases  >10% PH Cases |
| secondary hyperoxaluria [21] | malabsorptive syndromes, severe NC and ESRF, especially in Crohn’s disease and short bowel syndrome |  |  |  | |
| **4. Hyperuricosuria** | | | | | |
| Lesch-Nyhan syndrome [84] | normal at birth, then progressive psychomotor delay, gout, automutilation, hyperuricosuria with recurrent NL | urine alkalisation, allopurinol, rasburicase |  | male gender (x-chromosomal recessive) | |
| partial HPRT deficiency [85] | hyperuricosuria with wide spectrum of symptoms | urine alkalinization, allopurinol |  |  | |
| glycogenosis type 1a [86] | episodic severe hypoglycemia, lactic acidemia, hyperuricosuria, hypercalciuria, hypocitraturia, Fanconi syndrome (see Dent 1) recurrent NC, NL and CRF, renal amyloidosis | oral citrate supplementation, avoid sodium salts, thiazides, allopurinol |  |  | |
| **5.** **Hypouricosuria** |  |  |  |  | |
| APRT deficiency [22] | Urinary accumulation of the insoluble purine 2,8 dihydroxyadenine, NL and CRF | urine alkalisation, allopurinol, febuxostat |  | characteristic brownish round crystals | |
| xanthinuria [23] | low levels of uric acid in both serum and urine, xanthinuria, UL | increased fluid uptake |  | urine sediment with characteristic orange-brown color | |
| urate transporter 1 [87] | hyporuricemia, risk of exercise induced acute renal failure |  |  |  | |
| **6.** **Renal tubular acidosis (RTA)** | | | | | |
| RTA type 1 [25] | distal RTA‘, metabolic acidosis due to impaired H^+^ excretion, hearing loss, failure to thrive, rickets, hypokalemia, hypocitraturia, hypercalciuria, early NC, NL | alkali supplementation with sodium/potassium bicarbonate or citrate salts |  | usually higher doses of bicarbonate required, adapt to serum pH | |
| RTA type 2 [25] | proximal RTA, (milder) metabolic acidosis due to bicarbonate wasting, hypokalemia, growth retardation, ocular abnormalities, enamel defects, intellectual impairment, less severe hypercalciuria and hypocitraturia | “ |  |  | |
| RTA type 3 ‘mixed type‘ [25] | Bicarbonate wasting and inability to acidify the urine: RTA plus osteoporosis (Guibaud-Vainsel syndrome), cerebral calcifications, growth failure, intellectual impairment, conductive deafness | “ |  |  | |

Abbreviations: ACE: angiotensin converting enzyme; CaSR: calcium-sensing receptor; CRF: chronic renal failure, ESRF: end-stage renal failure; FS: Fanconi syndrome; NC: nephrocalcinosis; NL: nephrolithiasis; PTH: parathormone; RTA: renal tubular acidosis; UL: urolithiasis
